# Role of High-Dose Adjuvant Chemotherapy Followed by Autologous Stem Cell Transplantation in Locally Advanced Triple-Negative Breast Cancer: A Retrospective Chart Review

**DOI:** 10.1155/2022/3472324

**Published:** 2022-09-30

**Authors:** Bayan Al-Share, Hadeel Assad, Judith Abrams, Abhinav Deol, Asif Alavi, Dipenkumar Modi, Andrew Kin, Voravit Ratanatharathorn, Joseph Uberti, Lois Ayash

**Affiliations:** Department of Oncology, Wayne State University/Karmanos Cancer Institute, Detroit, MI, USA

## Abstract

**Purpose:**

Women with locally advanced/high-risk triple-negative breast cancer treated with the current standard chemotherapy continue to have a poor prognosis. High-dose chemotherapy with autologous stem cell transplant as treatment for locally advanced/high-risk breast cancer remains controversial due to a lack of survival benefit seen in previous phase III trials. However, these trials evaluated a heterogeneous group of patients with different receptor subtypes. A marginal benefit was observed in certain subgroups. We report long-term outcomes of women with stage IIB or III triple-negative breast cancer treated with high-dose chemotherapy followed by autologous stem cell transplant at our institution between 1995 and 2001.

**Methods:**

This is a retrospective analysis of stage IIB or stage III triple-negative breast cancer treated with high-dose chemotherapy followed by autologous stem cell transplant. We excluded women with hormone-positive, HER2/neu-positive/unknown, and/or metastatic disease prior to transplant as per updated AJCC 7^th^ edition guidelines. Patients underwent surgery and either neoadjuvant or adjuvant anthracycline and taxane-based chemotherapy and then proceeded to high-dose chemotherapy and autologous stem cell transplant using carmustine 600 mg/sqm, cyclophosphamide 5.6gm/sqm, and cisplatin 165 mg/sqm (STAMP 1 regimen) for consolidation. This was followed by locoregional breast and lymph node radiation per standard of care.

**Results:**

Twenty-nine women (2 stage IIB and 27 stage III) were evaluated. The median age at diagnosis was 43 years (IQR: 40, 51). Eleven patients had 4–9 regional lymph nodes (LN) involved and 16 had 10+ involved LNs. Four patients had T4 or inflammatory breast cancer and two had ipsilateral supraclavicular LNs involved. The median follow-up time is 16 years (95% CI: 12, 19, range <1–19 y) posttransplant. The median overall survival was 15 years (95% CI: 3, 19); the median DFS was 14 years (95% CI: 1, 19).

**Conclusions:**

This study of locally advanced/high-risk triple-negative breast cancer treated with adjuvant high-dose chemotherapy and autologous stem cell transplant reveals high overall survival rate. With the current improvement in treatment-related mortality, re-evaluating this approach in this subset of high-risk breast cancer in prospective randomized studies may be worthwhile.

## 1. Introduction

Triple-negative breast cancer (TNBC), first described in the mid-2000s, accounts for 15–20% of all breast cancer cases diagnosed worldwide. Patients with TNBC often are young (<40 years of age), premenopausal, African American, have more visceral metastases, and are carriers of pathogenic *BRCA* variants (particularly BRCA 1) [1–4]. The American Society of Clinical Oncology/College of American Pathology (ASCO/CAP) guidelines defined TNBC as negative estrogen and progesterone receptors (ER, PR) reflected by <1% expression by immunohistochemistry (IHC) and negative HER2/neu expression reflected by 0-1+ by IHC or 2+ by IHC with nonamplified fluorescence in situ hybridization (FISH) [5, 6].

At the genetic level, TNBC is heterogeneous. Lehmann et al. characterized six subtypes of TNBC based on molecular profiles [7]. Each subtype (basal-like 1, basal-like 2, immunomodulatory, mesenchymal, mesenchymal stem-like, and luminal androgen receptor) exhibited different sensitivities to therapeutic agents (e.g., basal-like 1 responds preferentially to cisplatin). Basal-like phenotypes are hypothesized to be more sensitive to agents that induce DNA double-strand breaks (cisplatin) or by synthetic lethality with agents that knock out two repair pathways (PARP inhibitors) [8]. Despite the higher chemosensitivity seen in some subtypes, the prognosis is worse for the whole TNBC population, with a sharp decrease in overall survival (OS) in the first three to five years after diagnosis [9].

Adjuvant high-dose chemotherapy (HDC) with autologous stem cell transplant (ASCT) for the treatment of breast cancer was introduced through multiple studies in the 1980s and 1990s [10]. Phase II clinical trials in the 1990s treated women with locally advanced/high-risk breast cancer and showed favorable outcomes [11, 12]. Therefore, it became part of the routine management of locally advanced/high-risk breast cancer in multiple centers worldwide during that period. Subsequent randomized phase III trials and a meta-analysis of 15 trials failed to confirm overall survival benefit but revealed improved recurrence-free survival [13–15]. Patient follow-up in these initial publications was generally short. Subgroup analysis showed a significant improvement in overall survival in the HER2-negative cases, with the greatest benefit among the triple-negative breast cancer group, for whom there was a 33% reduction in the risk of death [16].

The STAMP 1 regimen, composed of carmustine, cyclophosphamide, and cisplatin, was employed in multiple studies of autologous transplants in breast cancer [11, 17, 18]. A retrospective review of 443 patients treated with this regimen showed a low incidence of cardiotoxicity [19], in contrast to anthracycline-based regimens commonly utilized in TNBC. The enhanced response of TNBC to platinum-based chemotherapy makes this an attractive marrow ablative regimen for this breast cancer subtype. We performed a retrospective review of women with high-risk breast cancer who received STAMP I followed by autologous hematopoietic stem cell support at our institution. The long-term follow-up and efficacy data reveal a potential role for high-dose chemotherapy in TNBC.

## 2. Methods

### 2.1. Patient Population

The study included women with locally advanced TNBC (stages IIB, IIIA, IIIB, or IIIC) and with an ECOG performance status of 0-1. Disease stage was determined by surgical pathology for those who underwent upfront surgery or clinically for those who received neoadjuvant chemotherapy as per the updated AJCC 7^th^ edition [20]. All 29 patients met the definition of locally advanced/high-risk disease, with T4 or inflammatory cancer, and/or at least four regional lymph nodes involved at diagnosis. Demographic and clinical characteristics are described in Table 1.

### 2.2. Treatment

Patients were treated on multiple clinical trial protocols of high-dose chemotherapy and autologous stem cell transplant with minimal variations. The general treatment protocol was similar and included the following.

#### 2.2.1. Pretransplant Chemotherapy

Patients received 3-4 cycles of chemotherapy utilizing a combination of anthracycline and taxane-based regimens. Four patients received neoadjuvant chemotherapy. G-CSF was started 1–3 days after each cycle for primary neutropenia prevention.

#### 2.2.2. Bone Marrow Harvest and Leukapheresis

The source of autologous stem cells was dictated by the study protocol the patient was enrolled in. An autologous marrow harvest was obtained after the third cycle of induction chemotherapy. Peripheral blood progenitor cell collection was done after 5 days of G-CSF injections and required 3-4 h leukapheresis over 2-3 subsequent days depending on the study protocol.

#### 2.2.3. Intensification or HDC

Fourteen days after the last leukapheresis, patients were admitted and received high-dose chemotherapy consisting of cyclophosphamide (1,875 mg/m^2^/d) over 1 hour for 3 days (days 6, 5, and 4), cisplatin (55 mg/m^2^/d) administered as continuous infusion over 72 hours (days 6, 5, and 4), and carmustine (600 mg/m^2^) administered the day after completion of the other two agents (day 3). To prevent hemorrhagic cystitis caused by cyclophosphamide bladder toxicity, patients received aggressive hydration with at least 3 L per day of oral fluid or equivalent amount of IV fluid with replacing ml by ml of urine output, bladder irrigation, and mesna infusion. Infection prophylaxis with either trimethoprim/sulfamethoxazole or norfloxacin was started after completion of chemotherapy. Bone marrow or peripheral blood progenitor cells were infused 72 hours after completion of chemotherapy, followed by G-CSF administration until absolute neutrophil count of ≥1000/mm^3^ for 2 consecutive days was achieved.

#### 2.2.4. Radiation Therapy

All patients received locoregional treatment with radiation therapy delivered to the whole breast or chest wall and regional lymph nodes that started no sooner than six weeks after HDC and ASCT.

### 2.3. Statistical Analysis

The primary endpoint was overall survival (OS) defined from the date of bone marrow transplant to the date of death or last follow-up if death is not recorded; the secondary endpoint was disease-free survival (DFS), defined from the date of bone marrow transplant to date of signs or symptoms of disease or death, whichever occurred first, or last follow-up if neither signs of disease nor death were recorded. Treatment-related mortality was defined as death within 100 days of a transplant from any cause other than disease relapse. Time to engraftment was defined as time to hematologic reconstitution, measured from day 0 to the recovery of an absolute neutrophil count of ≥500/microliter, a platelet count >20 × 10^9^/L, and a hematocrit ≥25%, independent of transfusions.

OS and DFS were calculated using Kaplan–Meier methods with pointwise 95% confidence intervals around the medians. We estimated follow-up time using reverse Kaplan–Meier methods.

## 3. Results

### 3.1. Patient Characteristics

Twenty-nine women with confirmed TNBC were treated with HDC and ASCT between 1995 and 2001 (Table 1). The median age was 43 years, and most participants (83%) were European Americans. Most had stage III disease: six IIIA, four IIIB, and seventeen IIIC. Two participants had stage II disease.

Twenty-five women underwent upfront surgery followed by adjuvant anthracycline and taxane-based combination chemotherapy prior to HDC with ASCT. Four patients received neoadjuvant chemotherapy utilizing similar induction chemotherapy regimens followed by surgery and then HDC and ASCT. The most common surgery was modified radical mastectomy (69%). All participants were node-positive, and more than half (16/29) had 10 or more positive nodes on surgical pathology. Among the four patients treated with neoadjuvant therapy, three had pathology reports that showed residual disease: The first had a residual 9.5 cm tumor and one out of 27 positive axillary lymph nodes (yp T3N1Mx), the second had had a 4 cm residual tumor and eight out of nine positive axillary lymph nodes (yp T2N2Mx), and the third had tumor cells identified predominantly as dermal angiolymphatic tumor cell emboli with greater than 95% tumor regression after neoadjuvant chemotherapy and thirteen out of fourteen lymph nodes involved by cancer. The fourth patient's pathology report from surgery was missing.

Among all patients, two had clinical evidence of residual disease prior to autologous stem cell collection (one had a biopsy-confirmed residual chest wall nodule and one had a residual axillary lymph node on a CT scan). These two patients did not receive any additional anticancer therapy prior to the stem cell transplant. The median time from diagnosis to transplant was 5 months (95% CI 5, 6).

### 3.2. Treatment Toxicity

There was no transplant-related mortality within the first 100 days after transplant. There was no reported pulmonary or hepatic toxicity, veno-occlusive disease, grade 3 or more of hematologic toxicity or mucositis. Two patients died of viral encephalitis in the first year; both occurred seven months after transplant. One patient died of pneumonitis of unknown etiology 12 years after transplant. One patient died of capecitabine toxicity when received as a subsequent treatment after the patient relapsed.

### 3.3. Treatment Outcomes

The median follow-up for overall survival was 16 years (95% CI: 12, 19). All living participants have been followed for at least nine years. At the time of last the follow-up, seventeen of the participants had died: eight died of breast cancer, one died of capecitabine toxicity used in a subsequent line of therapy upon disease relapse, seven died of other causes, and one had an unknown cause of death (Table 2). In the first year, four patients (14%) died: two died of disease relapse and two of viral encephalitis.

**Figure 1:**
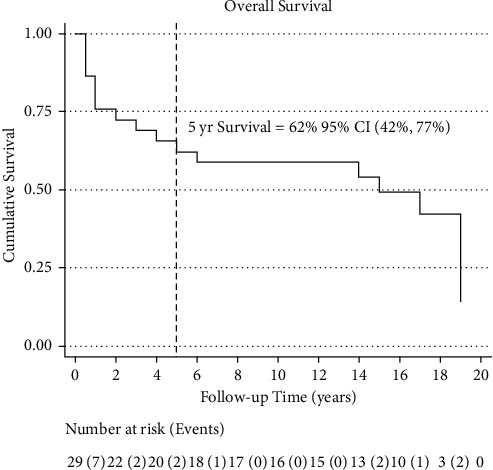
Overall survival. Kaplan–Meier estimates of overall survival (OS) time from date of bone marrow transplant to date of death or in case the participant did not die, the date of the last follow-up. Five-year OS is calculated from the Nelson–Aalen estimated cumulative (integrated) hazard function. It is denoted by the dashed line intersecting the axis at 5 years.

**Figure 2:**
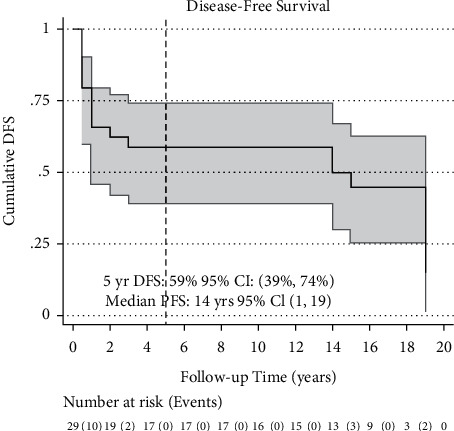
Disease-free survival. Kaplan–Meier estimate of disease-free survival (DFS) time from date of bone marrow transplant to date of recurrence or death, in case the participant's disease did not progress and the participant was still alive, date of the last follow-up. The five-year DFS is calculated from the Nelson–Aalen estimated cumulative (integrated) hazard function. It is denoted by the dashed line intersecting the X-axis at 5 years.

**Figure 3:**
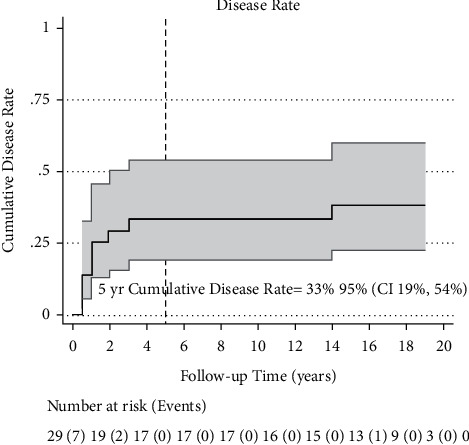
Cumulative disease rates of Kaplan–Meier graph. Kaplan–Meier estimates cumulative disease rate. Five-year time to recurrence DFS is calculated from the Nelson–Aalen estimated cumulative (integrated) hazard function. It is denoted by the dashed line intersecting the X-axis at 5 years.

The median overall survival was 15 years (95% CI: 3, 19) (Figure 1). Ten patients had disease recurrence; the median DFS was 14 years (95% CI: 1, 19) (Figure 2). Five-year OS and DFS were 62% (95% CI: 42%, 72%) and 59% (95% CI: 39%, 74%), respectively. The five-year recurrence rate was 33% (95 CI: 19%, 54%) (Figure 3).

Data on subsequent lines of therapy after disease recurrence were limited, however, included brain radiation (2 patients), taxol (1 patient), intrathecal methotrexate (1 patient with leptomeningeal disease), and capecitabine (1 patient).

## 4. Discussion

To our knowledge, this is the first study reporting long-term survival outcomes in locally advanced/high-risk TNBC with a median follow-up of 16 years. Our retrospective review of patients with locally advanced triple-negative breast cancer treated with high-dose chemotherapy followed by autologous hematopoietic stem cell transplant demonstrates an excellent median OS of 15 years. It also demonstrates the safety of the STAMP I regimen when overseen by practitioners experienced in recognizing and treating regimen-related toxicities.

Breast cancer is considered a systemic disease with evidence of micrometastasis demonstrated by circulating tumor cells early in the course of the disease [21]. The metastatic disease remains the most common underlying cause of death. Systemic treatments to prevent metastasis remain ineffective in a subset of patients with high-risk diseases manifested by hormone negativity and a high burden of lymph node involvement. Other treatment strategies including immunotherapy have limited activity and have not shown improvement in OS in the treatment of early-stage disease. Different treatment strategies are needed. Current standard chemotherapy regimens used for TNBC typically include a combination of anthracycline and taxane-based polychemotherapy [22–24]. The Anthracycline in Early Breast Cancer (ABC) trials revealed the superiority of anthracycline compared to taxane-based chemotherapy regimens in early-stage HR-positive and TN breast cancers [25]. The 4-year invasive disease-free survival (IDFS) for HR-negative subtype who had ≥4 LNs involved was 71.8% and 60.8% in the TaxAC and TC regimen, respectively [25]. The median IDFS was not reached. However, only a minority of these patients met the high-risk TNBC definition used in our study and total follow-up was relatively short. Initial studies also showed that combinations that used higher cumulative doses of anthracyclines were more effective (RR, 0.80; 95% CI: 0.72, 0.93), however, at the price of additional toxicity [26].

We reported a 5-year OS of 62% (95% CI: 42%, 72%) and a 5-year DFS of 59% (95% CI: 39, 74%) in patients with high risk for distant recurrence, defined as having a triple-negative disease with T4 tumors and/or greater than 4 positive LNs. All study participants received high-dose chemotherapy followed by autologous hematopoietic stem cell transplant in addition to definitive standard breast cancer treatment, suggesting that the benefit could be as a result of their intensified treatment regimen. Furthermore, the 5-year recurrence rate was 33%, with very few recurrences occurring after 5 years, consistent with existing literature on TNBC recurrence patterns.

Outcome data regarding the efficacy of high-dose chemotherapy with stem cell support in TNBC are sparse. The West German Study Group trial WSG AM-01 was the first trial to report significant survival benefits for women with >9 positive axillary nodes receiving high-dose chemotherapy using epirubicin 90 mg/m^2^, cyclophosphamide 3000 mg/m^2^, and thiotepa 400 mg/m^2^ (E_90_ C_3000_Thio_400_ q3 weeks x2 cycles) compared with dose-dense chemotherapy using epirubicin 90 mg/m^2^, methotrexate 40 mg/m^2^, and fluorouracil 600 mg/m^2^ (E_90_ M_40_F_600_ q2 weeks x3 cycles) [27]. For the whole group, the 5-year EFS (62% *vs.* 41%) and OS (76% *vs.* 61%) favored the high-dose arm. The most pronounced effect of high-dose chemotherapy was observed in the subgroup with TNBC. The 66 women with TNBC were younger (median age 45 years) and had higher grade tumors. For the 30 women with TNBC receiving high-dose chemotherapy, the median EFS was not reached, whereas the EFS was only 32.3 months for the 36 women receiving dose-dense chemotherapy.

An Italian National Registry study reviewed outcomes in 1183 women with >3 positive axillary nodes receiving adjuvant high-dose chemotherapy followed by ASCT (73% received a single alkylating agent-based regimen; 27% received epirubicin- or mitoxantrone containing multi-transplant regimens) [28]. For the whole group, the median DFS was estimated at 101 months and the median OS was at 134 months. The OS was significantly better in endocrine-responsive tumors and for those receiving multiple transplants. HER2 status did not affect survival. For the 85 patients with TNBC, the median OS was 110 months. The EBMT registry performed a retrospective analysis of 583 patients with >3 positive axillary nodes receiving adjuvant high-dose chemotherapy followed by ASCT between 1995 and 2005 [29]. With a median follow-up of 120 months, the 10-year DFS and OS were 44% and 58%, respectively. The OS was significantly better in endocrine-responsive tumors, those with <10 positive axillary nodes, and those with smaller (<2 cm) tumors. HER2 status did not affect survival. For the 35 patients with TNBC, the 10-year DFS and OS were 37% and 52%, respectively. Finally, an abstract presented in 2018 at the ESMO Congress in Munich from the Netherlands described 20-year data from a phase III trial conducted between 1993 and 1999, which randomly assigned 885 women with 4+ axillary nodes to conventional chemotherapy (epirubicin, cyclophosphamide, and fluorouracil) or the same regimen but replacing the last cycle with high-dose chemotherapy with stem cell support (cyclophosphamide, thiotepa, and carboplatin) [30]. For the whole population, there was no difference in survival or relapse. For those with >9 positive axillary nodes, however, high-dose chemotherapy significantly improved relapse-free survival (39% *vs.* 27%) and overall survival (44% *vs*. 30%). There was a trend in benefit for the TNBC cohort, with RFS of 51% and 34% (HR 0.66, *p*=0.07) and OS of 52% and 39% (HR 0.71, *p*=0.14), with high-dose and conventional chemotherapy, respectively. To our knowledge, there is an ongoing clinical trial evaluating high-dose chemotherapy with autologous stem cell transplant in triple-negative breast cancers as adjuvant therapy (ClinicalTrials.gov: NCT02670109).

The participants of our study were treated prior to the use of capecitabine in the postneoadjuvant setting in early-stage disease. CreateX (also known as JBCRG-04) trial studied the use of adjuvant capecitabine therapy in stage I–IIIB HER2-negative breast cancer patients who had residual tumors after receiving neoadjuvant anthracycline and taxane-based chemotherapy [31]. The primary endpoint of the study was DFS, which was longer in the capecitabine group compared to placebo in the whole population (5-year DFS was 74.1% *vs.* 67.6%). Almost one-third of patients in this trial had a triple-negative disease. Those who had capecitabine had a 5-year DFS of 69.8% compared to 56.1% in the placebo group [31].

Postneoadjuvant platinum therapy was compared to capecitabine in stage II-III TNBC patients with at least 1 cm of residual disease in the ECOG-ACRIN-EA1131 study, which did not demonstrate superiority or noninferiority of platinum therapy to capecitabine and was associated with higher toxicity in those who received platinum [32]. Immunotherapy has recently developed a role in the treatment of early-stage TNBC. The addition of pembrolizumab to neoadjuvant/adjuvant chemotherapy in stage II-III TNBC revealed higher pathologic complete response rates compared to placebo-chemotherapy (68.4% *vs.* 51.2%). At a median follow-up of 15.5 months, fewer patients had disease relapse in the pembrolizumab-chemotherapy group (7.4% *vs.* 11.8%) [33]. Results of the prespecified analysis of Keynote 522 showed a statistically significant improvement in EFS with pembrolizumab with a 36-month EFS rate of 84.5% *vs.* 76.8% [34].

Despite neoadjuvant or adjuvant therapy, there are patients who will still relapse and are considered chemoresistant to standard doses, where HDC followed by ASCT will fit in the era of adjuvant capecitabine and immunotherapy is unclear. However, studies investigating sequential or combination therapy with HDC and ASCT might be worthwhile.

Limitations to our study include the following: since this is a retrospective review of our database long after the time of transplant, we were unable to obtain treatment-related morbidity data if it was not documented in follow-up notes. This is a review of the BMT database from a single center, so the number of patients who met the criteria of locally advanced/high-risk triple-negative breast cancer was limited, although their survival data are important to report. HER2 testing was not performed routinely prior to 2010, and therefore, many patients were excluded due to unknown HER2 status, thus limiting our sample size. Last, our participants were treated in a clinical trial and had excellent performance status, which may limit the generalizability of the results to patients that would not otherwise be clinical trial candidates.

In conclusion, high-dose chemotherapy followed by ASCT provides a durable remission and survival in a subset of patients with locally advanced/high-risk triple-negative breast cancer. Triple-negative breast cancer continues to have the worst survival among other types of breast cancer despite recent advancements in treatment strategies. Re-evaluation of HDC with autologous stem cell support in this subset of high-risk breast cancer in prospective randomized studies may be worthwhile.

## Figures and Tables

**Table 1:** Patients' characteristics.

	*N* = 29
Age at BMT	
Median age in years (IQR)	43 (40–51)

Race	
Black	5 (17%)
European American	24 (83%)

Stage at diagnosis	
IIB	2 (7%)
IIIA	6 (21%)
IIIB	4 (14%)
IIIC	17 (59%)

Surgery	
Lumpectomy + LN evaluation	9 (31%)
Radical mastectomy	20 (69%)

Chemotherapy	
Adjuvant	25 (86%)
Neoadjuvant	4 (14%)

Positive nodes	
Fewer than 4	1 (3%)
4–9	11 (38%)
10 or more	16 (55%)
Missing^∗^	1 (3%)

BMT, bone marrow transplant; LN, lymph node. Number of positive nodes was determined by the surgical pathology and is the posttreatment pathological nodal stage for those that received neoadjuvant chemotherapy. ^∗^This patient was staged as IIIB and received neoadjuvant therapy, which indicates the cancer was inflammatory or may have spread to the internal mammary node.

**Table 2:** Treatment outcomes.

	Total *N* = 29
Progressed	
No	19 (66%)
Yes	10 (34%)

Site of recurrence	
Axillary lymph nodes	5 (17%)
Others^∗^	2 (7%)
Unknown	3 (10%)

Status	
Alive	12 (41%)
Deceased	17 (59%)

Cause of death	
Breast cancer	8 (28%)
Treatment (*x*)	1 (3%)
Others (*t*)	8 (31%)

^∗^Other sites of recurrence include breast, lung, supraclavicular, and infraclavicular lymph nodes. (*x*) Treatment-related death was due to capecitabine related toxicity. (*t*) Other causes of death include cardiovascular disease (2), liver failure (1), stroke (1), tick-borne viral encephalitis (2), vocal cord paralysis (1), and pneumonitis (1).

## Data Availability

The data used to support this study are available from the corresponding author upon request.

## Additional Points

This retrospective review is done to look for the possible benefit of high-dose chemotherapy with autologous stem cell support in a highly aggressive breast cancer type, triple-negative breast cancer. We have a long follow-up on patient survival that has not been reported before. The benefit of high-dose chemotherapy in breast cancer is controversial. However, our data show potential benefits in this subtype of breast cancer that lacks targeted therapy and is known to have a worse prognosis compared to other breast cancer subtypes. This study of locally advanced triple-negative breast cancer treated with adjuvant high-dose chemotherapy and autologous stem cell transplant reveals high overall survival. Re-evaluation of this approach in this subset of high-risk breast cancer in prospective randomized studies may be worthwhile.
